# A Comparative Evaluation of Computed Tomography Images for the Classification of Spirometric Severity of the Chronic Obstructive Pulmonary Disease with Deep Learning

**DOI:** 10.3390/diagnostics11060929

**Published:** 2021-05-21

**Authors:** Hiroyuki Sugimori, Kaoruko Shimizu, Hironi Makita, Masaru Suzuki, Satoshi Konno

**Affiliations:** 1 Faculty of Health Sciences, Hokkaido University, Sapporo 060-0812, Japan; sugimori@hs.hokudai.ac.jp; 2 Department of Respiratory Medicine, Faculty of Medicine, Hokkaido University, Sapporo 060-8648, Japan; hiron@hokkaido.med.or.jp (H.M.); suzumasa@med.hokudai.ac.jp (M.S.); satkonno@med.hokudai.ac.jp (S.K.); 3 Hokkaido Medical Research Institute for Respiratory Diseases, Sapporo 064-0807, Japan

**Keywords:** image classification, chronic obstructive pulmonary disease, deep learning

## Abstract

Recently, deep learning applications in medical imaging have been widely applied. However, whether it is sufficient to simply input the entire image or whether it is necessary to preprocess the setting of the supervised image has not been sufficiently studied. This study aimed to create a classifier trained with and without preprocessing for the Global Initiative for Chronic Obstructive Lung Disease (GOLD) classification using CT images and to evaluate the classification accuracy of the GOLD classification by confusion matrix. According to former GOLD 0, GOLD 1, GOLD 2, and GOLD 3 or 4, eighty patients were divided into four groups (*n* = 20). The classification models were created by the transfer learning of the ResNet50 network architecture. The created models were evaluated by confusion matrix and AUC. Moreover, the rearranged confusion matrix for former stages 0 and ≥1 was evaluated by the same procedure. The AUCs of original and threshold images for the four-class analysis were 0.61 ± 0.13 and 0.64 ± 0.10, respectively, and the AUCs for the two classifications of former GOLD 0 and GOLD ≥ 1 were 0.64 ± 0.06 and 0.68 ± 0.12, respectively. In the two-class classification by threshold image, recall and precision were over 0.8 in GOLD ≥ 1, and in the McNemar–Bowker test, there was some symmetry. The results suggest that the preprocessed threshold image can be possibly used as a screening tool for GOLD classification without pulmonary function tests, rather than inputting the normal image into the convolutional neural network (CNN) for CT image learning.

## 1. Introduction

Chronic obstructive pulmonary disease (COPD) affects an estimated 384 million people worldwide [1] and is reported to be the third leading cause of death worldwide [2]. The exacerbation of COPD not only leads to a significant decline in lung function [3] but also may shorten life expectancy and increase the risk of death [4,5], as well as causing significant economic and social burden [6]. COPD is defined by the Global Initiative for Chronic Obstructive Lung Disease (GOLD) published by WHO and the National Heart, Lung, and Blood Institute (NHLBI) [7] and has been revised since then. It is important for the early diagnosis of COPD to perform spirometry to measure forced expiratory volume in 1 s (FEV_1_)/forced vital capacity (FVC). In particular, there is an abundance of studies [8,9,10,11,12,13,14] using CT, which have reported important findings on the loss of lung function, progression, and prognosis over time. However, in patients with clinical findings or contact history suspected of recent COVID-19 infection, pulmonary function tests may be discontinued or postponed, and there is concern that performing procedures such as deep breathing or forced expiration with maximal effort may cause the spread of contaminated droplets and aerosols to the surrounding area, resulting in the spread of infection. Therefore, we believe that it is important to provide diagnostic assistance without using pulmonary function tests. In the conventional diagnosis of emphysema in COPD with significant emphysema lesions, the lesion is seen on CT as a low attenuation area (LAA) [15,16], and the boundary is characterized by the absence of a clear wall. In early emphysema, small clusters of LAAs are formed, but as the lesion progresses, the fused LAAs occupy most of the lung field; the Goddard method [17] is representative for visual emphysema severity classification on CT. Additionally, in COPD with significant peripheral airway involvement, which is thought to be one of the main loci in the pathogenesis of COPD together with emphysema, it has been pointed out that central airways are also involved [18]. There are many reports on CT imaging in emphysema including COPD [10,11,12,13,14,19], and CT imaging is widely used as a simple imaging modality to obtain information on the whole lung.

Recently, the deep learning application in medical imaging has been actively promoted, and in the field of medicine, it has been widely used for image classification [20,21,22,23], object detection [24,25,26], semantic segmentation [27,28,29,30], and so on and has been widely applied from disease classification to detection. Deep learning has been reported for the automatic classification of emphysema patterns in CT images [31], but CT images were used as a simple input without any preprocessing. In image classification using convolutional neural networks (CNNs), features are extracted from the entire image and classified, but whether it is sufficient to simply input the entire image or whether preprocessing is required to set up a supervised image has not been sufficiently investigated. Additionally, there has been no attempt to classify the spirometric severity of COPD using CT images based on the diagnosis results of pulmonary function tests using image classification technology of artificial intelligence. This study aimed to create a classifier trained with and without preprocessing to detect the groups that require therapeutic intervention using CT images and to evaluate the recall, precision, overall accuracy, and AUC of different spirometric classifications using confusion matrix.

## 2. Materials and Methods

### 2.1. Subjects

A total of eighty patients (male: 77; female: 3, mean age ± SD: 69.8 ± 7.9 years) who underwent a chest CT scan on a 4-row CT (SOMATOM plus Volume Zoom; Siemens AG, Berlin, Germany) between 2003 and 2006 were classified into four spirometric classifications: without airflow limitation (FVC ≥ 70%, former GOLD 0) (*n* = 20, 66.4 ± 7.2 years), GOLD 1 (*n* = 20, 67.3 ± 9.0 years), GOLD 2 (*n* = 20, 72.5 ± 6.8 years), and GOLD 3 or 4 (*n* = 20, 72.4 ± 7.2 years) based on the values of post-bronchodilator pulmonary function tests by using a rolling seal Chestac-33 spirometer (Chest MI, Tokyo, Japan) on the same day as CT scans. The pulmonary function, based on the values of post-bronchodilator spirometry carbon monoxide diffusing capacity (DL_CO_) and transfer coefficient (Kco), measured using the single breath method, was measured. The procedures met the requirements of the Japanese Respiratory Society Guidelines [32]. These subjects (Table 1) were participants in the Hokkaido COPD cohort study [33,34]. These subjects were included in this study because it is important to classify them using a group of subjects whose patient background has been diagnosed with accurate examinations in order to define accurate supervised images.

### 2.2. Preprocessing for the Lung CT Image

Two types of image data were created: the original image, which was converted from a DICOM image to a JPEG image with a pixel size of 512 × 512, and the threshold image, which was created by preprocessing a CT image to extract only CT values of −950 or less in the lung field and filling it with red. The threshold setting of −950 for the CT value was defined based on the report [35] that the threshold of −950 HU for the CT value defining emphysema has the best correlation with pathological emphysematous lesions. For the next step, since CT lung field images include the entire chest, including areas where lung fields are not depicted, based on the positional relationship between the trachea and both lung fields, we defined the lower lung field as the area where the upper border of the liver was not depicted and the upper lung field as the area where the trachea was not depicted anterior to the lung field. Based on the original image, slices were selected for the threshold image in the same procedure. These slices were selected based on the original image and the threshold image. With the above two types of preprocessing, we divided the data into training and test data sets and created four subsets to create a four-class classifier classified by stage so that fourfold cross validation could be performed (Table 2).

### 2.3. Training and Evaluation for Creating Models

**Figure 1:**
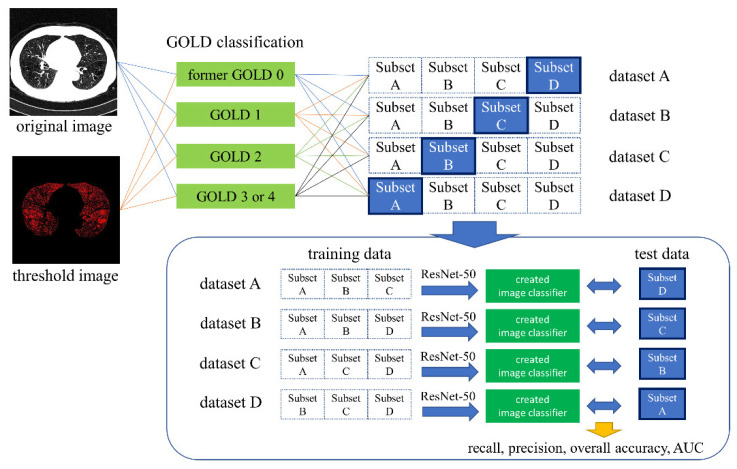
Schematic of the datasets and subsets for training and test.

**Figure 2:**
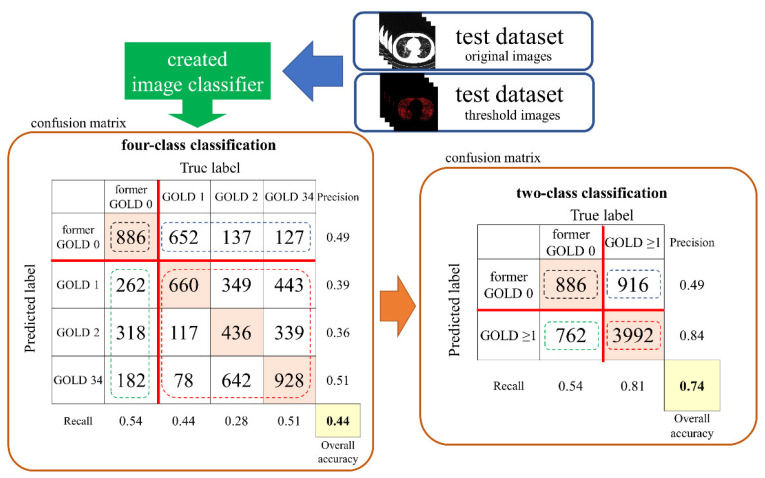
Confusion matrix for evaluating the recall, precision, and overall accuracy.

The software for the deep learning technique was developed with in-house MATLAB software (The MathWorks, Inc., Natick, MA, USA) and the use of a desktop computer with an NVIDIA RTX 1080Ti or RTX 2080Ti graphics card (Nvidia Corporation, Santa Clara, CA, USA). ResNet50 was used as the CNN, and the hyperparameters were set to epoch 10 and base learning rate 0.0001. The training dataset consisting of each subset was used for data augmentation, and the images were rotated from −25 degrees to 25 degrees. To create four classifiers for both the original image and the threshold image, we trained each of these images. The classifiers were tested with test data for each dataset, and the results of classification into former GOLD 0, GOLD 1, GOLD 2, and GOLD 3 or 4 were combined into a confusion matrix to obtain the recall, precision, overall accuracy, and AUC (Figure 1). The confusion matrix was further divided into two classes, former GOLD 0 and GOLD ≥ 1, and recall, precision, overall accuracy, and AUC were calculated for each class (Figure 2). The training was performed twice using RTX 1080Ti and RTX 2080Ti GPUs.

### 2.4. Statistical Analysis

The obtained recall, precision, overall accuracy, and AUC were expressed as mean ± standard deviation. The average of the calculation results on two GPUs per data set was used as the obtained results. The recall, precision, overall accuracy, and AUC were evaluated for each preprocessing. Firstly, we applied the Shapiro–Wilk test to the differences: if normality was accepted, the *t*-test was used; otherwise, the Wilcoxon signed ranked test was used. For comparing the methods of preprocessing, multiple comparisons between the spirometric classifications were performed to determine the optimal classification model. Levene’s homoscedasticity test was performed for the dose-dependence study. If homoscedasticity was confirmed, Dunnett’s multiple comparison test was used to assess between-group differences, while if homoscedasticity was not confirmed, the Steel–Dwass multiple comparison test was used. For comparing the methods of preprocessing, the McNemar–Bowker test was used to assess the symmetry of the confusion matrix. Differences in all statistical analyses were considered statistically significant if *p* was less than 0.05. All statistical analyses were performed using JMP software (version 14; SAS Institute Inc., Cary, NC, USA).

## 3. Results

### 3.1. Four-Class Analysis

The detailed results are shown in Table 3. The recall of original and threshold images was 0.35 ± 0.15 and 0.39 ± 0.17, respectively. The precision of original and threshold images was 0.36 ± 0.13 and 0.4 ± 0.12, respectively. The AUC for original and threshold images was 0.61 ± 0.13 and 0.64 ± 0.10, respectively. There were no significant differences in the mean values for recall, precision, overall accuracy, and AUC.

A comparison between the original and threshed images for each stage showed that, for the recall for the original image, there was a significant difference between GOLD 1 and GOLD 3 or 4 (*p* = 0.0202) and between GOLD 2 and GOLD 3 or 4 (*p* = 0.0052). For the precision for the original image, there was a significant difference between GOLD 1 and GOLD 3 or 4 (*p* = 0.0202). For the precision for the threshold image, there was a significant difference between GOLD 1 and GOLD 3 or 4 (*p* = 0.0074). There were no significant differences in the recall for the threshold image. For the AUC for the original image, there was a significant difference between GOLD 2 and GOLD 3 or 4 (*p* = 0.0052). For the AUC for the threshold image, there was a significant difference between GOLD 1 and GOLD 3 or 4 (*p* = 0.0202) and between GOLD 2 and GOLD 3 or 4 (*p* = 0.0372). In all datasets, the McNemar–Bowker test showed that the *p* value was less than 0.05.

### 3.2. Two-Class Analysis

The detailed results are shown in Table 4. Both recall and precision were significantly higher for GOLD ≥ 1, and symmetry was observed for dataset C in the McNemar–Bowker test (*p* = 0.5185).

## 4. Discussion

### 4.1. Statement of Principal Findings

In this study, we developed and evaluated a classifier for spirometric classification using CT images with and without preprocessing prior to image learning, and the highest recall, precision, and AUC values were found in GOLD 3 or 4. As the percentage of emphysema in the lungs increases in severe COPD, the feature changes due to the enlarged alveolar spaces in the original image captured the feature values in CNN due to the destruction of alveolar septa and alveolar architecture. In the threshed image, due to the coarsening of the filled area of the airway caused by the threshold setting of CT value −950, the features were described. This is also true for the results of former GOLD 0 or GOLD ≥ 1, and we comprehended that the results of GOLD 1 and above capture the structural changes caused by emphysema as a feature compared to former GOLD 0. Although there was no significant difference in this study, threshold images showed higher values than original images in all the result indices. The normal images are considered as positive data in CNN, which showed larger pixel values of lung structures including alveoli and arteriovenous veins than air. On the other hand, in the threshold image, features were learned from the positive data of the so-called air data, which were the data below the CT value of −950 in the thorax. Although both images in this study have a negative-positive relationship with each other, in the image classification, the results captured the features of each spirometric classification. In the normal image, the body contour information was used as positive data, and the weights and biases were updated in the CNN, so it was possible that features other than lung structure were also calculated. In this respect, the results of the original image were lower than those of the threshold image. Additionally, the air in the lung field was used as positive data to obtain feature values; however, in the actual image data, the thickening of the airway wall was ignored in the preprocessing. Airway alterations from central to small airways, besides emphysema, contribute to airflow limitation in COPD [18]. In the current study, we focused on the preprocessing of the lung field on inspiratory CT for the initial attempt to perform deep learning in respiratory diseases. It is plausible that learning about airway structures using inspiratory CT or the combined use of inspiratory and expiratory CT may improve the accuracy of the classification of spirometric severity. Although there was no direct relationship between severe COPD exacerbation and visual structure on CT images [9], another report showed that quantitative assessment using fractal exponent D detected the changes associated with exacerbations [36]. Moreover, the lung condition depending on the severity of the disease is characterized on CT images [8], and the image classification by deep learning can be used for the spirometirc severity classification. Additionally, in the two-class classification by threshold image, the recall and precision are over 0.8 in GOLD ≥ 1, and symmetry is observed in dataset C in the McNemar–Bowker test. Additionally, the symmetry of dataset C in the McNemar–Bowker test shows that the preprocessed threshold image can be used as a screening tool for spirometric classification without pulmonary function tests.

### 4.2. Strengths and Weaknesses of the Study

There are some limitations to this study. The first limitation was that the images were evaluated on a per image basis rather than on a per patient basis. However, in this study, all the images from the upper to lower lung fields of the patients in each spirometirc classification were given to the CNN as training data and were fed to the CNN as training data. Therefore, in mild cases such as GOLD 1, emphysema may not be present in the entire lung field, and when evaluated with a single image, an image equivalent to former GOLD 0 was also trained as GOLD 1. However, the creation of a dataset based on the diagnosed spirometric severity conducted in this study learns features even for regions that cannot be easily determined by humans, and it cannot necessarily be determined that the image is inappropriate. Secondly, the number of subjects was small. In this study, 20 subjects in each group were used. In addition, the subjects of this study were patients who entered the COPD cohort study and were classified according to the GOLD classification within cohort study. We simply compared the results with and without preprocessing using a fixed model of CT imaging to understand how the presence of preprocessing affects the accuracy of the classifier. Since medical images are said to have different image characteristics depending on the vendor and imaging conditions [37], it was necessary to minimize the effect of differences in conditions caused by using images from other facilities on classification accuracy. Since COPD is a systemic inflammatory disease with various complications, it is desirable to manage COPD to maintain the long-term activity of daily living using drug therapy and rehabilitation, especially in Japan, where the population is superaged. Inhalation drug clinical trials have shown that the decline in GOLD 2 is more effective than that in the advanced stage, suggesting that CT imaging can be used to detect patients who require therapeutic intervention.

## 5. Conclusions

In this study, we developed a classifier trained with and without preprocessing for spirometric classification using CT images and evaluated the recall, precision, overall accuracy, and AUC of different spirometric classifications by confusion matrix. In the two-class classification by threshold image, recall and precision exceeded 0.8 for GOLD ≥ 1, and in the McNemar–Bowker test, there was some symmetry. The results suggest that preprocessed threshold images can be used as a possible screening tool for spirometric classification without pulmonary function tests.

## Figures and Tables

**Table 1:** Characteristics of subjects.

	Former GOLD 0 (N = 20)	GOLD 1 (N = 20)	GOLD 2 (N = 20)	GOLD 3 or 4 (N = 20)
Age, yr (Mean ± S.D.)	66.4 ± 7.2	67.3 ± 9.0	72.5 ± 6.8	72.4 ± 7.2
Female sex, N (%)	1 (5)	2 (10)	0	0
Body mass index, kg/m^2^ (Mean ± S.D.)	22.9 ± 3.6	23.5 ± 2.6	22.8 ± 3.7	21.9 ± 2.4
Current smoker, N (%)	8 (40)	4 (20)	7 (35)	2 (10)
Smoking index, pack-years [Median (Q1-Q3)]	23.9 (45–86)	20.3 (43.2–65.3)	29.2 (46.7–69.8)	19.3 (53.5–76)
Lung function				
Post-bronchodilator				
FVC, % predicted (Mean ± S.D.)	99.8 ± 10.1	119.9 ± 10.9	100.7 ± 12.2	88.2 ± 13.2
FEV_1_, % predicted (Mean ± S.D.)	91.8 ± 13.7	90.2 ± 9.8	61.3 ± 6	40.4 ± 7.5
FEV_1_/FVC (Mean ± S.D.)	0.75 ± 0.08	0.61 ± 0.07	0.49 ± 0.08	0.37 ± 0.07
Reversibility of FEV_1_, % [Median (Q1-Q3)]	4.1 (0.2–6.1)	8.8 (1.6–7.2)	12.2 (4.9–23.1)	12.9 (8.8–27.3)
Reversibility of FEV_1_, ml [Median (Q1-Q3)]	96.6 (5–150)	137.6 (40–162.5)	135 (77.5–277.5)	108.2 (67.5–225)
DL_CO_, mmol/min/mm Hg (Mean ± S.D.)	90.9 ± 16.5	86.7 ± 18.1	84.1 ± 17.5	81.3 ± 29.9
Kco, mmol/min/mm Hg/L (Mean ± S.D.)	84.7 ± 20.2	75.6 ± 18.9	73.1 ± 19	74.6 ± 28.4

FVC: forced vital capacity, FEV_1_: forced expiratory volume in 1 sec, DL_CO_: carbon monoxide diffusing capacity, Kco: transfer coefficient, S.D.: standard deviation, Q1: first quartile, Q3: third quartile.

**Table 2:** The number of images in each subset.

	Subset A	Subset B	Subset C	Subset D	Total
Former GOLD 0	1545	1643	1578	1648	6414
GOLD 1	1790	1659	1828	1507	6784
GOLD 2	1716	1665	1651	1564	6596
GOLD 3 or 4	1665	1678	1820	1837	7000
Total	6716	6645	6877	6556	26,794

**Table 3:** Recall, precisions, AUC, and overall accuracy for four-class analysis.

		Original Image	Threshold Image
Recall	Former GOLD 0	0.34 ± 0.15	0.4 ± 0.19
	GOLD 1	0.28 ± 0.12	0.42 ± 0.14
	GOLD 2	0.26 ± 0.12	0.25 ± 0.16
	GOLD 3 or 4	0.51 ± 0.09	0.48 ± 0.07
Precision	Former GOLD 0	0.39 ± 0.07	0.42 ± 0.14
	GOLD 1	0.26 ± 0.1	0.3 ± 0.06
	GOLD 2	0.31 ± 0.17	0.39 ± 0.1
	GOLD 3 or 4	0.46 ± 0.09	0.5 ± 0.11
AUC	Former GOLD 0	0.64 ± 0.06	0.68 ± 0.12
	GOLD 1	0.56 ± 0.13	0.57 ± 0.08
	GOLD 2	0.50 ± 0.15	0.60 ± 0.07
	GOLD 3 or 4	0.72 ± 0.08	0.73 ± 0.07
Overall accuracy		0.35 ± 0.11	0.39 ± 0.06

**Table 4:** Recall, precisions, AUC, and overall accuracy for two-class analysis.

		Original Image	Threshold Image
Recall	Former GOLD 0	0.34 ± 0.15	0.40 ± 0.19
	GOLD ≥ 1	0.84 ± 0.06	0.84 ± 0.02
Precision	Former GOLD 0	0.39 ± 0.07	0.42 ± 0.14
	GOLD ≥ 1	0.80 ± 0.02	0.82 ± 0.04
AUC		0.64 ± 0.06	0.68 ± 0.12
Overall accuracy		0.72 ± 0.03	0.74 ± 0.04

## Data Availability

The created models in this study are available on request from the corresponding author. However, the image datasets presented in this study are not publicly available due to ethical reasons, e.g., containing information that could compromise the privacy of research participants.
